# Abattoir-based serological surveillance for transboundary and zoonotic diseases in cattle and swine in Cambodia: a pilot study in Phnom Penh province during 2019 and 2020

**DOI:** 10.1007/s11250-022-03309-1

**Published:** 2022-09-23

**Authors:** Jarunee Siengsanan-Lamont, Sothyra Tum, Lida Kong, Paul W. Selleck, Laurence J. Gleeson, Stuart D. Blacksell

**Affiliations:** 1 Mahidol-Oxford Tropical Medicine Research Unit, Faculty of Tropical Medicine, Mahidol University, Bangkok, Thailand; 2 National Animal Health and Production Research Institute, Phnom Penh, Cambodia; 3 Nuffield Department of Medicine, Centre for Tropical Medicine & Global Health, University of Oxford, Oxford, UK

**Keywords:** Zoonoses, Animal disease surveillance, Cambodia, ASF, CSF, PRRS, Brucellosis, Q fever

## Abstract

A pilot animal disease surveillance program was implemented at four abattoirs in Phnom Penh, Cambodia, between October 2019 and January 2020. A total of 1141 samples were collected from 477 cattle and 664 swine. Serological testing was performed using commercial antibody ELISA kits for zoonotic and high-impact animal diseases, namely brucellosis, Q fever, classical swine fever (CSF), porcine reproductive and respiratory syndrome (PRRS) and African swine fever (ASF). Only two samples tested positive for *Brucella* antibodies (0.2%, 95% CI 0.4–0.6, *n* = 1141). The seroprevalence of Q fever was 0.8% (95% CI 0.3–2.1, *n* = 477) in the cattle samples, while CSF, PRRS and ASF in pigs were 55.4% (95% CI 51.6–59.2, *n* = 655), 81.2% (95% CI 78.1–84.0, *n* = 655) and 2.6% (95% CI 1.6–4.1, *n* = 664), respectively. All 38 doubtful and 17 positive ASF antibody ELISA samples were negative when tested by real-time PCR. Univariate analyses demonstrated that the factor significantly associated with positive results of ASF was the abattoir location (*p*-value = 0.002). Based on logistic regression models, significant risk factors for CSF were province of origin (*p*-value = 1.7 × 10^−6^), abattoir (*p*-value = 3.6 × 10^−11^) and PRRS positivity (*p*-value = 0.004), and for PRRS were province of origin (*p*-value = 0.0004) and CSF positivity (*p*-value = 0.001). In conclusion, the seroprevalences of zoonotic diseases in this study were very low. The high prevalence of CSF and PRRS antibodies were most likely the result of vaccination. All ASF seropositive pigs, including those that gave equivocal results, originated from large-scale Cambodian-based commercial farms, as well as Thailand, which raises questions about possible illegal vaccination or low-pathogenicity ASF variants. The pilot abattoir serological surveillance program described here has the potential to provide a sentinel for incursions of novel and endemic pathogens, although further work is required to demonstrate its capacity to provide information on the longitudinal disease trends.

**Supplementary Information:**

The online version contains supplementary material available at 10.1007/s11250-022-03309-1.

## Introduction

The Kingdom of Cambodia is a country with a population of approximately 16 million people, where livestock production provides livelihood, nutrition and food security (Holl, 2018). Meat consumption and animal production in Cambodia are increasing due to population and economic growth and urbanisation (Darith et al., 2017). Smallholder livestock raisers face threats from multiple endemic diseases including foot and mouth disease (FMD), haemorrhagic septicaemia, classical swine fever (CSF) (Shankar et al., 2012), porcine reproductive and respiratory syndrome (PRRS) (Goutard et al., 2015) and more recently African swine fever (ASF). For the past two decades, animal disease surveillance activities in Cambodia have focussed on emerging infectious diseases (EIDs) including highly pathogenic avian influenza (Desvaux et al., 2006; Ear, 2012; Horm et al., 2013) with few studies on surveillance of other high-impact transboundary animal diseases (Tum et al., 2015; Vergne et al., 2012). There is also limited surveillance information on brucellosis (Sothoeun et al., 2013) and Q fever, two potentially important zoonoses.

Cambodia has limited human resources, diagnostic capacity and capability and has relied on international agencies to support animal disease surveillance activities (Desvaux et al., 2006; Goutard et al., 2015). At present, there is no routine surveillance program to compile animal disease information, although there was national interest to trial a readily applicable surveillance program to fill this gap. The surveillance program described in this study was initiated by the Cambodian government, and the testing was performed with the guidance of Mahidol-Oxford Tropical Medicine Research Unit (MORU) staff at the National Animal Health and Production Research Institute (NAHPRI) located in Phnom Penh. The objectives of the program were to determine seroprevalences of endemic high-impact diseases, to strengthen national capability in disease surveillance and detection and to develop a sustainable animal disease surveillance system. The outcomes from this study were expected to contribute to the knowledge of disease prevalence in Cambodian livestock and further improvement of disease control measures and livestock production in the country.

## Material and methods

### Site selection and sample size calculation

For this trial, four abattoirs in Phnom Penh Municipality, namely Boeng Salang (processing swine and cattle), Chrouy Changva (processing cattle only), Damnak Thum (processing swine only) and Trea Boun (processing swine only) slaughterhouses, were selected based on high numbers of livestock processed per day and convenient access. The Boeng Salang and Chrouy Changva slaughterhouses are relatively large, processing around 300–400 and 120–150 cattle per day, respectively, while the other two facilities process less animals per day (Asia Beef Network, 2020) at around 200 pigs per night. Samples were collected between October 2019 and January 2020. The sample size calculation was based on Cannon and Roe’s technique using an expected prevalence of approximately 10% and test sensitivity of 90% (Cannon & Roe, 1982). Thus, the sample collection team (comprised of two NAHPRI staff, an abattoir veterinarian from the provincial office and a veterinarian from MORU) was directed to randomly collect at least 30 samples (or approximately 30% of the total number of animals slaughtered) on the collection day. Despite the study design planning to visit each abattoir once a month, abattoir visits were dependent upon local staff availability. Thus, each abattoir was visited four to five times in total, and intervals between visits were inconsistent. Information including the number and type of animals slaughtered at the abattoir on the sampling day and where known the biodata of the sampled animals (e.g. country, province or farm of origin, trader/owner, sex, age and vaccination status), were recorded.

### Sample preparation and storage

Blood samples were collected from the jugular vein of cattle and swine via jugular venopuncture and then transferred to labelled vacutainers without anticoagulants. The vacutainers were placed in a rack sealed in a large zip-lock plastic bag and kept in a cooler with freezer packs while being transported back to NAHPRI. Once the blood samples arrived at NAHPRI, serum was separated within 24 h using a refrigerated centrifuge. The serum (supernatant) was pipetted into a labelled microcentrifuge tube (or cryotube). The serum samples were stored at 2–8 °C while handling and kept at − 20 °C or lower in an allocated freezer for long-term storage.

### Laboratory diagnostic tests

Commercial enzyme-linked immunosorbent assay (ELISA) kits manufactured by IDvet,^1^ France were used to detect antibodies against *Coxiella burnetii* (ID Screen® Q fever indirect multi-species, Cat# FQS-MS-5P); *Brucella abortus*, *melitensis* or *suis* (ID Screen® brucellosis serum indirect multi-species, Cat# BRUS-MS-10P); PRRS virus (ID Screen® PRRS indirect, Cat# PRRSS-5P); CSF virus E2 glycoprotein (ID Screen® classical swine fever E2 competition, Cat# CSFE2C-5P); and ASF virus (ID Screen® African swine fever competition, Cat# ASFC-5P). ELISAs were performed according to the manufacturer’s protocols provided with the kits. Sample to positive ratio (S/P%) and competition percentage (S/N%) were calculated using IDSoft™ software provided with the ID Screen® ELISA kits (ID.VET, 2014). The cutoff, diagnostic sensitivities (Dse) and specificities (Dsp) of the brucellosis, Q fever, PRRS, CSF and ASF ELISA kits are presented in Table 1. Samples testing positive and doubtful positive for brucellosis antibodies ELISA were confirmed by the rose bengal test (RBT) (World Organization for Animal Health, 2021, 2022). Samples that tested positive and doubtful positive in the ASF antibody ELISA were then tested by real-time PCR conducted by NAHPRI as a part of their public diagnostic services using primers described by King et al. (2003).

**Table 1:** ELISA kit information

ELISA kit	Cutoff point	Dse/Dsp*
Brucellosis	S/P% ≥ 120% = positive 110% < S/P% > 120% = doubtful S/P% ≤ 110% = negative	100% / 99.7% (Changoluisa et al., 2019)
Q fever	S/P% > 50% = positive 40% < S/P% ≥ 50% = doubtful S/P% ≤ 40% = negative	100% / 100% (Changoluisa et al., 2019)
PRRS	S/P ≤ 0.4 = negative S/P > 0.4 = positive	n/a
CSF	S/N% ≤ 50% = positive 50% < S/N% ≤ 60% = doubtful S/N% > 60% = negative	n/a
ASF	S/N% ≤ 40% = positive 40% < S/N% < 50% = doubtful S/N% ≥ 50% = negative	95.8%/ 99.4% (CISA-INIA, 2015)

*Diagnostic sensitivity/diagnostic specificity

### Statistical analysis

Descriptive statistics, apparent seroprevalence, true prevalence (using Dse and Dsp published in previous papers), risk factor and spatial analyses were performed in Microsoft Excel (Microsoft Corporation, 2020) and R Studio Version 1.2.1335 (RStudio Team, 2020). Frequency and probability distributions were used to explain the dataset. Apparent and true seroprevalences were estimated applying the Wilson method suggested for imperfect tests (Reiczigel et al., 2010) using the epiR package (Stevenson, 2020). Given the low disease prevalence, Fisher’s exact test was used to determine the associations between a factor for seropositive and seronegative animals (Soetewey, 2020). The variables including sex, age, animal origin, origin province (or country), abattoir and seropositivity were tested against the ELISA results. As individual abattoirs in Phnom Penh sourced animals from different locations, the abattoir was considered one of the variables. Where applicable, univariate (Fisher’s exact or chi-square) and multivariate logistic regressions were fitted using the glm() function to identify potential risk factors. A subset of variables with a *p*-value < 0.1 in the univariate model were included in multivariate analyses (Souriya et al., 2020). The final model was selected based on the following: (1) all variables in the model had *p*-value < 0.05 tested by analyses of variance (ANOVA) chi-square test (University of Connecticut, 2020), and (2) the model had the lowest Akaike information criterion (AIC). The goodness of fit of the final model was tested using Hosmer–Lemeshow χ2 test (Anonymous, 2011). Odds ratios (OR) with 95% confidence intervals (CI) were calculated. Visualisation of animal movement data was generated using the leaflet R package (Graul, 2016).

## Results

A total of 1141 (477 cattle and 664 pigs) samples were collected during this period. The overall seroprevalence of diseases is given in Table 2, including apparent and true prevalence taking into account diagnostic sensitivities and diagnostic specificities. The numbers of animal samples collected per calendar month is presented in Table 3. The survey design suggested sampling ~ 30% of animals present at the abattoir on the sample collection days, so the numbers of samples collected on each visit varied depending on this factor. The average cost of field sample collection, including staff per diem, transportation and field equipment and consumables, was approximately USD 2 per sample, while the cost of the field consumables alone was approximately USD 0.5 per sample. The cost of a serological diagnostic test and laboratory consumables ranged between USD 0.8 and USD 3.8 per sample, depending on the pathogen.

**Table 2:** Overall serological test results

Type	Disease	Positive	%Apparent seroprevalence (95% CI)	%True seroprevalence (95% CI)
Cattle (*n* = 477)	Brucellosis	1	0.2 (0.0, 1.2)	-
	Q fever	4	0.8 (0.3, 2.1)	0.8 (0.3, 2.1)
Swine (*n* = 655)	Brucellosis	1	0.2 (0.0, 0.9)	-
	CSF	363	55.4 (51.6, 59.2)	55.4 (51.6, 59.2)
	PRRS	532	81.2 (78.1, 84.0)	81.2 (78.1, 84.0)
	ASF (*n* = 664*)	17	2.6 (1.6, 4.1)	2.1 (1.1, 3.6)

*Due to the small volume of 9 serum samples, the samples were only tested for ASF

**Table 3:** Monthly sample counts and apparent seroprevalence percentage of each disease

	Collection month				%Seroprevalence (95% CI)		
Species	Month	Total (*n*)	Brucellosis	Q fever	PRRS	CSF	ASF
Cattle	Nov 2019	10	0.0 (2 × 10^−15^, 27.8)	10.0 (0.5, 40.4)	n/a	n/a	n/a
	Dec 2019	181	0.0 (0, 2.1)	0.6 (0.0, 3.1)	n/a	n/a	n/a
	Jan 2020	286	0.3 (0.0, 2.0)	0.7 (0.2, 2.5)	n/a	n/a	n/a
Swine	Oct 2019	58	0.0 (0, 6.2)	n/a	72.4 (59.8, 82.2)	46.6 (34.3, 59.2)	3.4 (1.0, 11.7)
	Nov 2019	10	0.0 (2 × 10^−15^, 27.8)	n/a	0.0 (2 × 10^−15^, 27.8)	0.0 (2 × 10^−15^, 27.8)	0.0 (2 × 10^−15^, 27.8)
	Dec 2019	178	0.6 (0.0, 3.1)	n/a	70.8 (63.7, 77.0)	44.9 (37.8, 52.3)	4.5 (2.3, 8.6)
	Jan 2020	409	0.0 (0, 0.9)	n/a	89.0 (85.6, 91.7)	62.6 (57.8, 67.1)	1.7 (0.8, 3.4; *n* = 418*)

*Due to the small volume of 9 serum samples, the samples were only tested for ASF

**Fig. 1:**
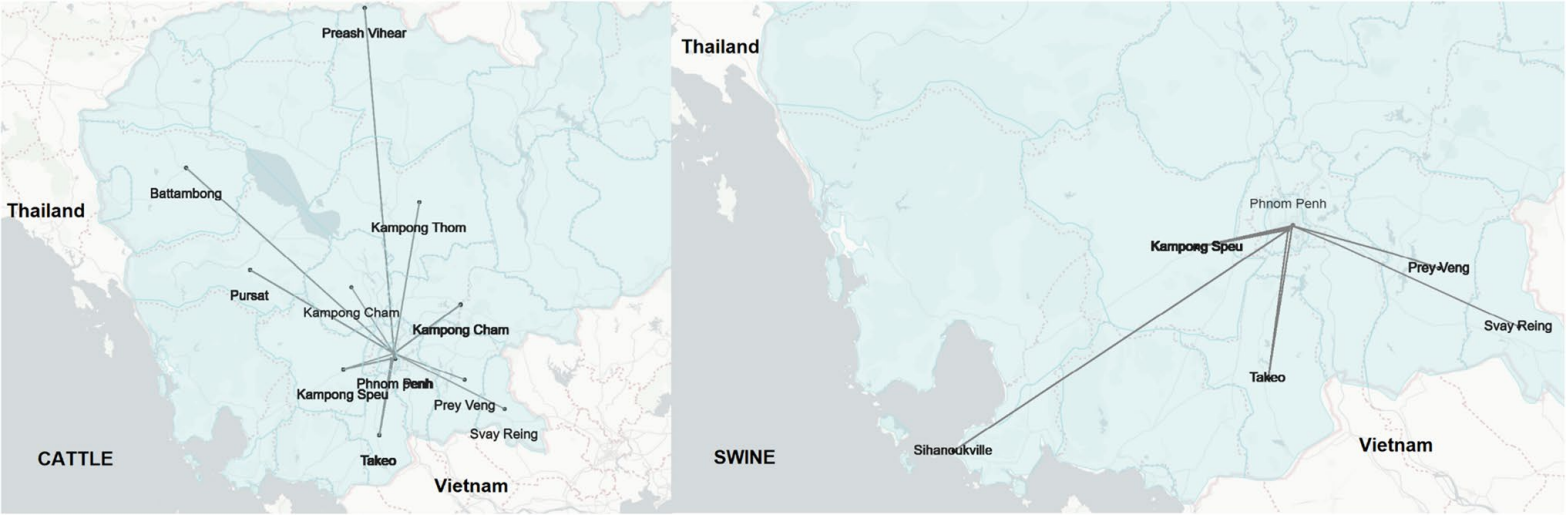
Movements of Cambodian cattle (left) and pigs (right) from province of origin to slaughterhouses in Phnom Penh (black dots and province names represent animal origins)

The origins of animals were plotted onto a map for visualisation of animal movements (Fig. 1). No vaccination history was recorded as animals were delivered to abattoirs by middlemen who did not have individual animal data. For cattle, 43.2% (*n* = 477) of the samples collected were from cattle imported from Thailand. For cattle of Cambodian origin, (*n* = 271), 41.1%, 14.3%. 12.5% and 10.7% were from Takeo, Kampong Cham, Pursat and Kampong Speu provinces, respectively. The age of cattle sampled ranged from 1 to 8 years old, with a majority of 28.5% (*n* = 477) aged 4 years old. All four *C. burnetii* seropositive cattle (0.83%, *n* = 477) were collected on the same day from the same abattoir with animals that originated from Takeo province. There were one cattle (0.2%, *n* = 477) and one pig (0.15%, *n* = 655) that tested positive for *Brucella* antibodies, and both originated from Takeo province. Only 23.7% (*n* = 664) of swine samples were pigs imported from Thailand (Table 4). The rest of the swine samples were from local animals from multiple provinces in Cambodia, with 92.9% (*n* = 506) from commercial pig production farms. However, the age of the abattoir-sampled pigs was not consistently recorded. Animal data were presented in the supplement table. Overall seroprevalence of CSF, PRRS and ASF were 55.4% (*n* = 655), 81.2% (*n* = 655) and 2.1% (*n* = 664) respectively. Seroprevalence of the Cambodian large commercial farm-origin pigs was 53.8% (*n* = 461) for CSF, 81.1% for PRRS (*n* = 461) and 3.0% for ASF (*n* = 470), while seroprevalence for the Thai-origin pigs (*n* = 158) for CSF, PRRS and ASF were 66.5%, 88.0% and 1.9%, respectively. Only 36 local swine samples were recorded as not from a large commercial farm with a seroprevalence of 27.8% for CSF, 52.8% for PRRS and 0% for ASF. Seropositive ASF samples were collected from pigs originating from Kampong Speu, Takeo and Sihanoukville provinces and Thailand. A total of 55 swine samples (38 equivocal and 17 positives to the ASF antibody ELISA) were negative when tested using real-time PCR to check for the presence of the ASF genomic material in the sample.

**Table 4:** Summary of swine sample seroprevalence results

Abattoir	Origin province	Total (*n*)	%Seroprevalence (95% CI)		
			CSF	PRRS	ASF
Boeng Salang	Kampong Speu	132	37.1 (29.4, 45.6)	78.8 (71.1, 84.9)	0.8 (0.0, 4.2)
	Prey Veng	9	44.4 (18.9, 73.3)	66.7 (35.4, 87.9)	0.0 (0, 29.9)
	Svay Reing	5	80.0 (37.6, 99.0)	80.0 (37.6, 99.0)	0.0 (3.1 × 10^−15^, 43.4)
	Takeo	20	10.0 (2.8, 30.1)	55.0 (34.2, 74.2)	0.0 (1.2 × 10^−15^, 16.1)
	Thailand	9	44.4 (18.9, 73.3)	77.8 (45.3, 93.7)	11.1 (0.6, 43.5)
Damnak Thum	Kampong Speu	125	53.6 (44.9, 62.1)	82.4 (74.8, 88.1)	5.2 (2.6, 10.4; *n* = 134*)
	Sihanoukville	31	48.4 (32.0, 65.2)	71.0 (53.4, 83.9)	9.7 (3.3, 24.9)
	Thailand	29	55.2 (37.5, 71.6)	89.7 (73.6, 96.4)	6.9 (1.9, 22.0)
Trea Boun	Kampong Speu	147	74.8 (67.2, 81.2)	86.4 (79.9, 91.0)	1.4 (0.4, 4.8)
	Takeo	18	27.8 (12.5, 50.9)	55.6 (33.7, 75.4)	5.6 (0.3, 25.8)
	Thailand	120	70.8 (62.1, 78.2)	88.3 (81.4, 92.9)	0.0 (0. 3.1)
	Unknown	10	20.0 (5.7, 51.0)	60.0 (31.3, 83.2)	0.0 (2 × 10^−15^, 27.8)

*Due to the small volume of 9 serum samples, the samples were only tested for ASF

Fisher’s exact method was used to determine the association of each of the recorded variables compared with antibody status for brucellosis, *C. burnetii* and ASF as the overall number of seropositive samples was low. The variable that demonstrated significant differences between positive and negative results for ASF the factor was the abattoir (*p*-value = 0.002). No factor was significantly different between positive and negative brucellosis (both cattle and swine) and *C. burnetii* (cattle) samples. For CSF and PRRS datasets, univariate (chi-square) and multivariate logistic regressions were fitted. Based on the final logistic regression models, significant risk factors for CSF were province of origin (*p*-value = 1.7 × 10^−6^), abattoir (*p*-value = 3.6 × 10^−11^) and PRRS positivity (*p*-value = 0.004) and for PRRS were province of origin (*p*-value = 0.0004) and CSF positivity (*p*-value = 0.001). The risk factors and its OR are presented in Table 5. Samples collected from Damnak Thum (OR = 1.9, 95% CI 1.2–3.0, *p*-value = 0.007) and Trea Boun (OR = 4.4, 95% CI 2.8–7.0, *p*-value = 1.3 × 10^−10^) abattoirs had significant higher CSF seroprevalences than Boeng Salang. Pigs originated from Takeo (OR = 0.2, 95% CI 0.1–0.4, *p*-value = 0.0001) and unknown (OR = 0.1, 95% CI 0.0–0.5, *p*-value = 0.005) province had significant lower CSF seroprevalences than pigs from other provinces. Positive PRRS pigs were more likely to test positive to CSF (OR = 1.9, 95% CI 1.2–2.9, *p*-value = 0.004). Similar to the CSF dataset, pigs originated from Takeo province (OR = 0.3, 95% CI 0.2–0.7, *p*-value = 0.002) had a lower PRRS seroprevalence than pigs from other provinces, and seropositive CSF results were significantly correlated to PRRS results (OR = 2.0, 95% CI 1.3–3.0, *p*-value = 0.001).

**Table 5:** Significant risk factors included in the final models

Variables/reference group	Odds ratio (95% CI)	*p*-value
**CSF dataset**		
Abattoir/Boeng Salang		
1. Damnak Thum	1.9 (1.2, 3.0)	0.007
2. NameTrea Boun	4.4 (2.8, 7.0)	1.3×10^−10^
Province of origin/Kampong Speu		
1. Takeo	0.2 (0.1, 0.4)	0.0001
2. Unknown	0.1 (0.02, 0.5)	0.005
PRRS ELISA test result/negative		
1. Positive	1.9 (1.2, 2.9)	0.004
**PRRS dataset**		
Province of origin/Kampong Speu		
2. Takeo	0.3 (0.2, 0.7)	0.002
CSF ELISA test result/negative		
3. Positive	2.0 (1.3, 3.0)	0.001

## Discussion

Animal health surveillance activities in Cambodia are limited, with only a small number of neglected parasitic diseases and FMD surveillance programs supported by the government. Other programs including influenza (Goutard et al., 2015; Osbjer et al., 2007), Japanese encephalitis, West Nile (Auerswald et al., 2020) and wildlife disease (Hul et al., 2021) surveys have been supported by international organisations and/or aid agencies. To support the animal health service to strengthen disease surveillance activities, our study chose abattoir-based surveillance over structured surveillance as it is simpler to implement in a low-resource setting. Even though disease information collected from an abattoir surveillance program has limitations and may not represent the true prevalence of diseases in the population (Cannon & Roe, 1982), it is still useful to provide an indication of the likely prevalence and, therefore, probable impact of diseases, as well as indications of geographic distribution, for further investigation. Important in this context, a study by Blacksell et al. (2008) also demonstrated that overall disease seropositivity determined by structured and abattoir surveys were relatively similar.

A major constraint identified during our study was that central staff from NAHPRI were well-trained and often facilitated field sample collections with little assistance from abattoir veterinarians, resulting in limited knowledge transfer and capacity building of on-site staff. Another constraint was the lack of laboratory capability and capacity due to inadequate human and financial resources. Capacity building of field provincial officers (i.e. abattoir veterinarians and animal health workers) in disease surveillance, sample collection, submission, case reporting and biosafety principles are critical for early detection and disease monitoring and control. Currently, many field investigations are performed by NAHPRI staff. The average cost of the field consumables per sample in Cambodia was half of the cost of those previously reported in a similar program in Lao PDR (Siengsanan-Lamont et al., 2021). Local supplies are widely available in Cambodia, resulting in lower costs of consumables. However, in looking at cost-effectiveness and sustainability, the cost of the diagnostic test kits must also be considered. In this instance, specific project resources supported the surveillance in order to obtain baseline indications of prevalence and to guide how to build a system for the longer term. The question arises as to the longer term utility of such a surveillance system. The system might be used from time to time to get a snapshot of the likely prevalence of priority diseases, especially zoonoses, in the livestock population. In a similar program in Lao PDR, the surveillance activity and associated training have also provided capacity building for field surveillance and laboratory diagnosis and was generally met with positive attitudes of field staff. Other approaches to conduct disease monitoring and surveillance like combining syndromic surveys using mobile phones or internet networks with case-by-case sample testing and/or field investigation should be explored.

The total numbers of samples collected per trip varied depending on the numbers of animals processed for slaughter on the sample collection days. Animals were often delivered to slaughterhouses by traders or delivery drivers who may or may not have had full records of individual animals but generally did not. Thus, some biodata, especially vaccination history, was not available, but if animals originated from outside the country, this was generally known. Interpretation of the results needs to take into account biases caused by these limitations of the sample and data collections. An interesting observation from the study was the significant numbers of animals of Thai origin processed in the Cambodian abattoirs, and previous studies have reported similar findings. Cambodia has previously been reported as a thoroughfare for cattle from Myanmar and Thailand to Vietnam and China (Pham et al., 2015). Declining cattle production in Cambodia coupled with an increased demand for animal protein (Olmo et al., 2017) has driven the importation of livestock, with the sample in this study revealing that a large proportion (~ 43%) of cattle processed in these slaughterhouses in Phnom Penh had come in from Thailand. Pisei (2020) reported that Cambodia imported around 20% (~ 2000–3000 live pigs/day) of its pigs from Thailand. Another study in 2012 reported that Cambodia imported pigs and cattle from Thailand and cattle from Vietnam (Kerr et al., 2013). However, our study had no record of animals at the abattoir from Vietnam. The absence of animals from Vietnam was likely due to the first ASF outbreak in Vietnam in February 2019 (Woonwong et al., 2020), at which time the Cambodian government banned the importation of pigs from Vietnam in March 2019 (Xuxin, 2019). There are three large-scale commercial piggery companies in Cambodia owned by multinational regional agribusiness companies (Pisei, 2020), which were the sources of most swine samples in our study. In May 2020, the General Directorate of Animal Health and Production at the Ministry of Agriculture, Forestry and Fisheries announced a reduction of live pig imports from neighbouring countries, mainly from Thailand, to 1800–2100 head per day and a prohibition of the transit of live pigs from Thailand to Vietnam, to help support local pig production (Chan, 2020). As demand for red meat continues to grow (Woonwong et al., 2020; Young et al., 2014), movements of animals and animal products continue to pose a risk for spreading transboundary animal diseases. It is a major challenge for Cambodian authorities to maintain meat supplies and at the same time prevent the movement of serious livestock diseases into the country. Currently, ASF poses a major threat to pig production in Cambodia, especially at the smallholder level in villages. Furthermore, lumpy skin disease (LSD) is also threatening the cattle population in the region as it has become widespread in China (Roche et al., 2020), and recent outbreaks have been reported in Thailand (Sripiachai, 2021). Abattoir surveillance might be useful to monitor the prevalence of endemic disease conditions or to quickly establish the distribution and impact of a recently introduced disease (e.g. PRRS) and it may help detect a new disease with less dramatic clinical manifestations, such as LSD.

In this study, the seroprevalence of Q fever and brucellosis in cattle was relatively low. In 2008, 120 cattle samples collected from six villages in three provinces in Cambodia tested negative to brucellosis by RBT (Sothoeun et al., 2013). Another study conducted in 2015 in Sa Kaeo province, Thailand (located close to the Thai-Cambodian border), reported that the herd-level seroprevalence of brucellosis and Q fever of beef cattle was 2.6% (95% CI 0.9, 7.3) and 4.3% (95% CI 1.8, 9.6) (Colombe et al., 2018). Brucellosis and Q fever are zoonoses and pose a public health risk, especially to people who are in close contact with infected animals (Mori & Roest, 2018). Further investigation in Takeo province, where seropositive animals originated, could provide more information on the disease distribution. It is interesting that both these diseases have a very low seroprevalence in Cambodia, where there are no control programs in place. In Cambodia, ruminants not only provided draught power but also meant extra income for rural households (Osbjer et al., 2015). The majority of cattle were kept in the small-scale system at home or nearby lands where pasture grazing was a common practice (Samkol et al., 2015). Another study by Colombe et al. (2018) reported the seroprevalence of brucellosis and Q fever in small ruminants at a Thai-Cambodia border community at 13.3% and 33.3%, respectively. Our surveillance did not collect small ruminant samples as these animals are commonly slaughtered at the household level or restaurants. As livestock lived in close proximity with humans, and high-risk behaviours such as consuming sick/dead animals were reported (Osbjer et al., 2015), monitoring of both zoonotic diseases in susceptible hosts would help better understand the disease risks in the human population.

Interpretation of the CSF and PRRS serology results is difficult as these abattoir-collected samples had no vaccination history, and vaccination programs for large commercial pig farms in Cambodia were not publicly available. Vaccines against CSF and PRRS are commonly used in pig production in South East Asia (Kunavongkrit & Heard, 2000; Thammakarn et al., 2018; Zhang et al., 2017) and are widely available in Cambodia. General Directorate of Animal Health and Production (GDAHP) reported in 2018 that Village Animal Health Workers (VAHWs) vaccinated up to 70% of backyard pigs (GDAHP, 2018). However, studies reported that vaccination rates of smallholder or backyard pigs, especially in rural areas, were lower. Despite government providing CSF vaccine for farmers free of charge (Wallberg, 2011), CSF and PRRS vaccine use in smallholders and semi-commercial farms were reportedly low (Sothoeun et al., 2013; Tornimbene et al., 2014; Tornimbene et al., 2015; Zhang et al., 2017). King (2016) reported that less than 50% of smallholder pigs were vaccinated against CSF, and vaccine failure was also a concern. The diagnostic kits used in our study could not differentiate antibodies arising post-vaccination from those arising post-infection. Thailand reported PRRS outbreaks in 2019 and 2020, while CSF outbreaks were reported in Northern Thailand only in 2019 (WOAH-WAHIS, 2022). No outbreaks of CSF and PRRS in Cambodia were reported between 2019 and 2020 (WOAH-WAHIS, 2022). Moreover, our study demonstrated a positive correlation between PRRS and CSF seropositivity. Thus, high seroprevalences of CSF and PRRS detected in our study most likely resulted from vaccine-induced antibodies. As more than 90% of the pig samples were from large commercial farms, Thai pigs showed higher seroprevalence than the Cambodian commercial pigs. Information on vaccination records and actual practices in commercial farms and smallholders would help explain the findings. The low numbers of smallholder pigs at abattoirs likely reflected population decline (due to competition from commercial piggeries and perhaps ASF outbreaks) and cultural practices where these pigs were often slaughtered at the household level.

For cattle, the province of origin variable was not significantly correlated with the seropositive *C. burnetii* samples despite the fact that all four positive samples originated from Takeo. For pigs, risk factor analyses suggested that province of origin, abattoir and seropositive CSF and PRRS statuses were potential predictors of serological reactors. However, there is not enough information at this stage to conclude the role these factors play. In the case of ASF tests, only the abattoir variable was significantly correlated to seropositive samples. Further investigation in the areas where positive animals originated from would provide more in-depth disease information. The first confirmed ASF cases were reported in Ratanakiri province in April 2019, and then five other provinces close to the Vietnam border were later confirmed ASF outbreaks (FAO, 2020). Control measures implemented by the Cambodian government included movement controls on live pigs and pig products and stamping out in the affected areas (FAO, 2020). Scientific publications on ASF control in Cambodia were not available at the time of our study. All ASF antibody positive and doubtful samples were from three Cambodian large commercial farms and also from Thailand, which most likely were commercial farm pigs. There was no indication of ASF in either of these production sources. Introduction of the currently circulating strain of ASF into commercial operations would be expected to result in high mortalities (FAO, 2020; Mazur-Panasiuk et al., 2019) and trigger some sort of alert, even if only in local media. ASF outbreaks were not reported in Cambodia (Pig Progress, 2022) during the time of our study. This raised some concerns as to the nature of the positive results. The Dse published by the ASF ELISA manufacturer’s internal validation was 95.8%, while the Dsp was reported up to 100% (Gallardo et al., 2014), indicating that false-negative but not false-positive results could occur. All the positive samples were confirmed in a retest to rule out test aberration on the days of testing, suggesting that these animals had been previously exposed to the virus. Samples that tested positive or doubtful by the antibody ELISA technique were negative when tested in the RT-PCR, indicating that they were not persistently infected survivors of infection. ASF is a highly contagious disease with a mortality rate of up to 100%, and a vaccine was not available. The results would indicate that under field conditions, the ASF ELISA has a Dsp of less than 100% unless there has been some use of vaccines that might have been imported from elsewhere. It is noted that commercial ASF vaccine is not available at the time of our study. However, there was a report on fake ASF vaccine productions in China since 2019 (Patton, 2021). Moreover, it is unlikely that an undetectable low virulence mutant (Pig Progress, 2021) would emerge in Cambodia. In general, each abattoir sorted animals from different sources. Thus, the abattoir factor was significantly associated with the ASF results. Interestingly, the origin province factor was not significant. This may be due to the denominators of the origin province being larger. Therefore, further investigations, especially those with positive results, are required to determine the true nature of this ASF seropositivity.

In conclusion, an abattoir surveillance system could provide initial disease seroprevalence of high-impact diseases and zoonoses for further investigation if needed. When resources are limited, the focus of the survey should be adjusted based on national priorities and the current situation. The cost-effectiveness of a survey program could be increased through building provincial veterinary and para-veterinary capacities and cost-cutting where possible.

## Supplementary Information

Below is the link to the electronic supplementary material.

Supplementary file1 (DOCX 15.5 KB)

## Data Availability

All data generated or analysed during this study are included in this publication.

## Footnotes

1 ID.VET: 310 rue Louis Pasteur, 34790 Grabels, FRANCE, https://www.id-vet.com/
