# Supplementary material for: Abattoir-based serological surveillance for transboundary and zoonotic diseases in cattle and swine in Cambodia: a pilot study in Phnom Penh province during 2019 and 2020
Source: Trop Anim Health Prod. 2022 Sep 23;54(5):316. doi: 10.1007/s11250-022-03309-1 (PMC9508002; doi:10.1007/s11250-022-03309-1)
Supplement: Supplementary file 1 — Supplementary file1 (DOCX 15.5 KB) [file 11250_2022_3309_MOESM1_ESM.docx]

| **Cattle** | | **N** |
| --- | --- | --- |
| Province of origin | Battambang | 11 |
|  | Kampong Cham | 39 |
|  | Kampong Chhnang | 3 |
|  | Kampong Speu | 29 |
|  | Kampong Thom | 8 |
|  | Phnom Penh | 16 |
|  | Preash Vihear | 12 |
|  | Prey Veng | 4 |
|  | Pursat | 34 |
|  | Svay Reing | 4 |
|  | Takeo | 111 |
|  | Thailand | 206 |
| Sex | F | 194 |
|  | M | 283 |
| Age | upto 1 Year | 5 |
|  | 1-2 Years | 139 |
|  | 2-3 Years | 106 |
|  | 3-4 Years | 139 |
|  | 4-5 Years | 69 |
|  | 6 Years and above | 19 |
| **Swine** | | **N** |
| Province of origin | Kampong Speu | 413 |
|  | Prey Veng | 9 |
|  | Sihanoukville | 31 |
|  | Svay Reing | 5 |
|  | Takeo | 38 |
|  | Thailand | 158 |
|  | Unknown | 10 |
| Sex | F | 420 |
|  | M | 244 |

Supplementary table 1: animal data
